# Novel Action of Carotenoids on Non-Alcoholic Fatty Liver Disease: Macrophage Polarization and Liver Homeostasis

**DOI:** 10.3390/nu8070391

**Published:** 2016-06-24

**Authors:** Yinhua Ni, Fen Zhuge, Mayumi Nagashimada, Tsuguhito Ota

**Affiliations:** Department of Cell Metabolism and Nutrition, Brain/Liver Interface Medicine Research Center, Kanazawa University, Kanazawa, Ishikawa 920-8640, Japan; shali0145@gmail.com (Y.N.); zgf0725@gmail.com (F.Z.); nakanaga@staff.kanazawa-u.ac.jp (M.N.)

**Keywords:** NAFLD/NASH, carotenoids, macrophages/Kupffer cells, insulin resistance, inflammation, fibrosis, antioxidant, β-cryptoxanthin, astaxanthin

## Abstract

Non-alcoholic fatty liver disease (NAFLD) is the most common chronic liver disease. It is characterized by a wide spectrum of hepatic changes, which may progress to non-alcoholic steatohepatitis (NASH) and cirrhosis. NAFLD is considered a hepatic manifestation of metabolic syndrome; however, mechanisms underlying the onset and progression of NAFLD are still unclear. Resident and recruited macrophages are key players in the homeostatic function of the liver and in the progression of NAFLD to NASH. Progress has been made in understanding the molecular mechanisms underlying the polarized activation of macrophages. New NAFLD therapies will likely involve modification of macrophage polarization by restraining M1 activation or driving M2 activation. Carotenoids are potent antioxidants and anti-inflammatory micronutrients that have been used to prevent and treat NAFLD. In addition to their antioxidative action, carotenoids can regulate macrophage polarization and thereby halt the progression of NASH. In this review, we summarize the molecular mechanisms of macrophage polarization and the function of liver macrophages/Kupffer cells in NAFLD. From our review, we propose that dietary carotenoids, such as β-cryptoxanthin and astaxanthin, be used to prevent or treat NAFLD through the regulation of macrophage polarization and liver homeostasis.

## 1. Introduction

**Figure 1:**
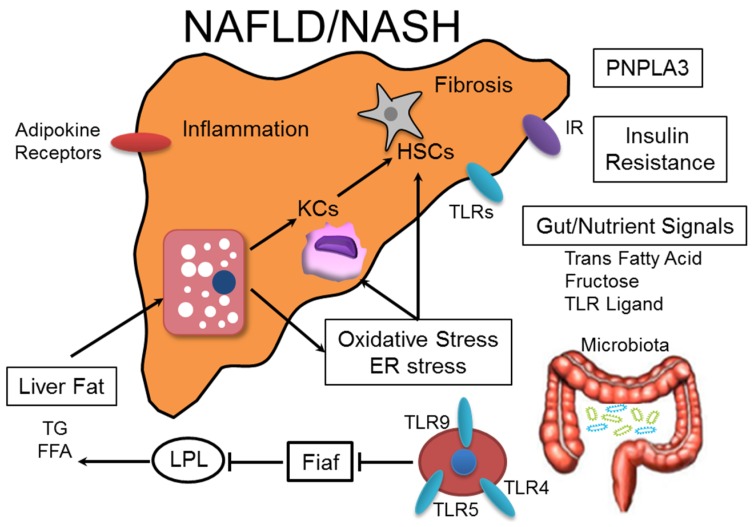
Multiple parallel-hit hypothesis of the progression of NAFLD/NASH. Overloading of lipids consisting primarily of triglycerides (TGs) and free fatty acids (FFAs) induces hepatic steatosis. Adipokines, such as interleukin (IL)-6 and tumor necrosis factor (TNF)-α, produced by adipocytes lead to hepatocyte fat accumulation and liver inflammation. The microbiota decreases epithelial expression of fasting-induced adipocyte factor (Fiaf), which functions as a circulating lipoprotein lipase (LPL) inhibitor and, therefore, is an important regulator of peripheral fat storage. Gut-derived signals can be affected by ingested trans fatty acids, fructose, or Toll-like receptor (TLR) ligands. Ingested FFAs and free cholesterol induce endoplasmic reticulum (ER) stress and oxidative stress, leading to hepatic inflammation and fibrogenesis. The presence of single nucleotide polymorphisms (SNPs) in the patatin-like phospholipase 3 (PNPLA3) gene increases the risk for NAFLD and NASH development across ethnicities.

Non-alcoholic fatty liver disease (NAFLD) is becoming increasingly prevalent. It is a common cause of chronic liver disease and a major indicator of metabolic syndrome [1]. Non-alcoholic steatohepatitis (NASH) is a more severe form of NAFLD that is broadly defined by the presence of steatosis with inflammation and progressive fibrosis [2], ultimately leading to cirrhosis and hepatocellular carcinoma (HCC) [3,4,5]. Multiple risk factors are associated with NAFLD, but the mechanisms underlying its onset and progression are still unclear. According to the initial “two-hit hypothesis”, insulin resistance is the “first hit” that leads to hepatic steatosis. The “second hit” is driven by oxidative stress, which leads to steatohepatitis and fibrosis [6]. In our previous studies, we found that insulin resistance promoted the progression of NASH from simple fatty liver [7]. Moreover, this traditional view has been developed within a more complex “multiple parallel-hit hypothesis”, which comprises a wide spectrum of parallel hits (Figure 1), including insulin resistance, oxidative stress, genetic and epigenetic mechanisms, environmental elements, cytokines, and microbiota changes [8].

Many cross-sectional clinical studies have been performed to elucidate the pathogenesis of NASH. However, due to the limitations of clinical studies, many researchers have focused on establishing animal models to assess the effect of different molecules on fatty liver formation. Dietary models of NASH are useful to mimic the pathogenesis of diet-induced obesity and its resulting metabolic disturbances, including NAFLD and NASH [9]. However, dietary methionine- and choline-deficient (MCD) mouse models experience severe weight loss and liver atrophy, which are not characteristics of NASH in human patients [10]. Similarly, a choline-deficient l-amino-acid (CDAA) diet causes weight loss and prevents insulin resistance [10]. Exclusive, long-term intake of a high-fat (HF) diet resulted in fatty liver and obesity in mice, but a HF diet over several months did not affect fibrosis [11]. Moreover, addition of trans fats or high levels of fat to a fructose diet promoted glucose and insulin insensitivity and fatty liver formation without fibrosis [12]. In our previous study, we found that a diet high in fat, cholesterol, and cholate led to the development of pathophysiological NASH in humans [13]. Furthermore, we found that excessive hepatic lipid accumulation promoted the activation of macrophages/Kupffer cells (KCs), leading to exacerbation of insulin resistance, hepatic inflammation and fibrogenesis [13].

Many pharmacotherapeutic strategies have been attempted in NASH, but there is no consensus on how to evaluate NASH patients or treat them using pharmacological therapies [14,15,16]. Recent randomized controlled trials have shown that insulin sensitizers, such as metformin or thiazolidinedione, do not improve liver histology significantly in NASH [17,18]. In the TONIC trial, both metformin and vitamin E did not lead to a sustained reduction in alanine aminotransferase (ALT) levels in children and adolescents with NAFLD. Although NASH resolution was greater in vitamin E-treated subjects, fibrosis was not improved [18]. In the PIVENS trial, pioglitazone improved steatosis and inflammation but led to significant weight gain. In contrast, compared with placebo, vitamin E improved liver enzyme levels and all histological features of NASH, except fibrosis [17]. Therefore, there is a clear need for additional therapies. So far, it remains unclear whether micronutrient antioxidant supplementation, particularly carotenoids, can be used to prevent and treat NAFLD.

Efforts have been made to understand the roles of immune cells, such as macrophages, natural killer cells, Th1/Th2 cells and T regulatory cells (T regs), in the pathogenesis of NASH and their potential therapeutic relevance. Specifically, hepatic macrophages, which consist of resident KCs and recruited bone marrow-derived macrophages, are the major cells that produce inflammatory mediators, such as tumor necrosis factor (TNF)-α and interleukin (IL)-1β, causing systemic insulin resistance and, ultimately, NASH [19]. In tissues, macrophages mature and acquire specialized functional phenotypes upon activation by different stimuli. In general, classical M1 activation is stimulated by Toll-like receptor (TLR) ligands, such as lipopolysaccharide (LPS) and interferon-gamma (IFN-γ), while alternative M2 activation is stimulated by IL-4/IL-13 [20,21]. Dysregulation and polarization of M1/M2 macrophages can lead to chronic inflammation, infection, cancer, obesity and its associated disorders, and NAFLD [21]. Recently, the protective effects of M2 macrophages/KCs were reported against alcoholic fatty liver disease and NAFLD by promoting M1 macrophage/KC apoptosis were reported [22]. Therefore, specific macrophage-targeted therapies are now starting to appear in the clinical arena. In particular, the reorienting and reshaping of macrophage polarization is extremely important in macrophage therapeutic targeting [23]. In this review, we discuss the involvement of hepatic macrophages/KCs on the pathogenesis of NASH and the impact of carotenoids on NAFLD prevention and treatment.

## 2. Micronutrients and NAFLD/NASH Management

The treatment of NAFLD patients should be based on a global approach, not only addressing insulin resistance and metabolic syndrome, but also including strategies focused on reducing oxidative stress, dyslipidemia, and cardiovascular risk. Apart from pharmacological therapies, the usual management of NAFLD includes lifestyle counseling to increase physical activity and achieve gradual weight reduction. Management of weight and overall fitness is the cornerstone of treatment for all patients with NAFLD. Several studies have demonstrated the benefit of weight loss in reducing steatosis or the NAFLD activity score on histology, with greater weight loss associated with more substantial improvements [24]. Reductions in ALT levels and steatosis occur even with small decreases in weight, whereas resolution of NASH, or even fibrosis, occurs with more marked or sustained weight loss, such as that observed after bariatric surgery [24,25,26,27]. However, weight loss through energy restriction is difficult to achieve and sustain [28]. Physical activity and exercise are also effective at decreasing steatosis. Cross-sectional and prospective studies have shown that physical activity decreases intrahepatic lipid levels [29]. Both aerobic and resistance exercises have been shown to improve liver function, independently of weight loss [30,31].

In addition to total energy intake, the composition of the diet also affects metabolic and endocrine functions, and overall energy balance in NAFLD patients [32]. General recommendations include reductions in the intake of total fat, saturated fatty acids, trans fatty acids, and fructose [33]. Most recommendations encourage the consumption of diets rich in fruits and vegetables for prevention of chronic disease, including NAFLD. Such diets would provide a significant amount of bioactive components with known beneficial effects due, in part, to their anti-inflammatory and anti-oxidative properties [32,33]. Therefore, these bioactive compounds or micronutrients, taken in combination with conventional treatments for cardio-metabolic diseases, may be used to prevent and treat NAFLD.

Several nutraceutical supplements have shown promising results as NAFLD treatments, especially those containing antioxidants and polyphenols, such as omega-3 fatty acids [34,35], anthocyanins, silymarin, and resveratrol [36]. However, results are derived mostly from animal studies and small trials with considerable heterogeneity with respect to inclusion criteria, sample size, type of experimental interventions and duration. Therefore, there are insufficient data to support or refute the use of these bioactive compounds to treat NAFLD patients. Further studies, especially large-scale, well-designed, randomized controlled trials, should be performed to verify the therapeutic effect of these compounds on NAFLD.

## 3. Liver Macrophages in NAFLD/NASH

Macrophages are an essential component of innate immunity and play a central role in inflammation and host defense [37]. Tissue-resident and -recruited macrophages are major players in the mechanisms of innate resistance and are links between inflammation and cancer [21,38]. In the liver, resident macrophages/KCs fulfill homeostatic functions, orchestrate tissue remodeling in ontogenesis, and regulate metabolic functions. KCs are strategically positioned in liver sinusoids, where they trap and phagocytose microbes in the circulation and act as the first line of resistance against blood-borne pathogens [21,38]. In addition, KCs have been found to interact with other immune cells involved in the pathogenesis of NAFLD [39]. Under steady-state conditions, KCs can inhibit dendritic cell-induced antigen-specific T cell activation and promote the suppressive activity of T regs. Upon activation by bacterial antigens, such as LPS, KCs modulate the differentiation and activation of various immune cells, including dendritic cells, T lymphocytes and neutrophils [40]. Resident and recruited cells of the monocyte-macrophage lineage exert a dual function in liver pathology [41,42]. Moreover, KC identification is based on the expression of F4/80 and CD11b and/or CD68. Liver F4/80^+^CD68^+^ macrophages are characteristic of resident KCs, which are phagocytic cells that produce reactive oxygen species. Cytokine-producing bone marrow-derived macrophages (BMDMs) express CD11b [43]. Compared with CD11b^+^ cells, CD68^+^ cells preferentially adhere to liver sinusoidal endothelial cells or hepatocytes [44,45].

Cells of the monocyte-macrophage lineage are characterized by considerable diversity and plasticity. In tissues, mononuclear phagocytes respond to environmental cues (e.g., microbial products, damaged cells and activated lymphocytes) with the acquisition of distinct functional phenotypes. In response to various signals, macrophages may undergo classical M1 activation (stimulated by TLR ligands and IFN-γ) or alternative M2 activation (stimulated by IL-4/IL-13) [46]. Plasticity and flexibility are key features of mononuclear phagocytes and of their activated states [21,38]. The phenotype of polarized M1/M2 macrophages can, to some extent, be reversed in vitro and in vivo [21,38]. Moreover, pathology is frequently associated with dynamic changes in macrophage activation, with classically-activated M1 cells implicated in initiating and sustaining inflammation, and M2 or M2-like cells associated with decreasing chronic inflammation [47].

### 3.1. Molecular Determinants of Macrophage Polarization

**Figure 2:**
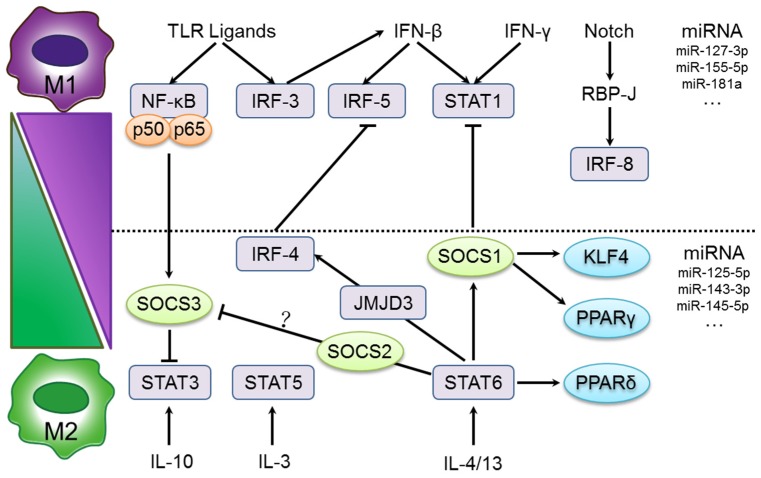
Mechanisms of macrophage polarization. The major pathways of macrophage polarization, which belong to the interferon regulatory factor (IRF)/signal transducer and activator of transcription (STAT)/suppressor of cytokine signaling (SOCS) (IRF-STAT-SOCS) families, are outlined. Cross-talk between SOCS/STAT and IRF components in M1 and M2 macrophage polarization is indicated. PPARγ and PPARδ control distinct aspects of M2 macrophage activation and oxidative metabolism. KLF4 participates in the promotion of M2 macrophage functions by cooperating with STAT6. IL-4 also induces the M2-polarizing Jmjd3-IRF4 axis to inhibit IRF5-mediated M1 polarization. IL-10 promotes M2 polarization through the induction of p50 NF-κB homodimers and STAT3 activities. MicroRNAs (miRNAs) have also emerged as critical regulators of macrophage polarization.

A network of signaling molecules, transcription factors, epigenetic mechanisms, and post-transcriptional regulators underlie the different forms of macrophage activation (Figure 2). Macrophage polarization is regulated by the interferon regulatory factor (IRF)/signal transducer and activator of transcription (STAT)/suppressor of cytokine signaling (SOCS) (IRF-STAT-SOCS) family of proteins. Canonical IRF/STAT signaling activation by IFN and TLR signaling induces a M1 macrophage phenotype through STAT1, and IL-4 and IL-13 signaling induces a M2 macrophage phenotype via STAT6 [21,48]. M1 macrophages upregulate IRF5, which is essential for the induction of cytokines (IL-12, IL-23 and TNF-α) involved in eliciting Th1 and Th17 responses [49]. The IL-4 type I and type II receptors activate Stat6 [37], which in turn activate the transcription of genes associated with M2 polarization including mannose receptor (Mrc1), resistin-like α (Retnla), and chitinase 3-like 3 (Chi3l3) [50]. IL-10 activates STAT3-mediated expression of genes (Il10, Tgfb1 and Mrc1) associated with a M2-like phenotype [51,52]. It has been suggested that IL-3-mediated STAT5 activation promotes M2 polarization [38]. IRF5 and IRF8 regulation through Notch are also part of the M1-associated transcriptional network [49,53].

Members of the SOCS family regulate STAT-mediated activation of macrophages. LPS or IFN-γ concert with TLR stimulation to upregulate SOCS1 and SOCS3 expression which, in turn, inhibit the activation of STAT1 and STAT3, respectively. On the other hand, upregulation of SOCS1 is crucial for IL-4-induced M2 characteristics [54,55]. A recent study showed that Notch signaling plays a critical role in the determination of M1 versus M2 polarization of macrophages, and compromised Notch pathway activation leads to a M2-like phenotype [56]. Furthermore, it was found that this effect is regulated in a SOCS3-dependent manner [56].

Additionally, downstream transcription factors also contribute to macrophage polarization. The nuclear receptors peroxisome proliferator activated receptor (PPAR)γ and PPARδ control a distinct subset of genes associated with M2 macrophage activation and oxidative metabolism [19,57,58]. Interestingly, Krüppel-like factor 4 (KLF4) cooperates with Stat6 to promote M2 gene transcription (Arg-1, Mrc1, Fizz1, and PPARγ) and inhibit M1 gene transcription (Tnf-α, Cox-2, Ccl (C-C chemokine ligand) 5, and iNOS) via sequestration of coactivators required for nuclear factor kappa-light-chain-enhancer of activated B cells’ (NF-κB) activation [59]. KLF2 regulates macrophage activation by inhibiting NF-κB/hypoxia-inducible factor (HIF)-1α activity [60]. IL-4 also induces c-Myc activity in human macrophages, which controls genes involved in M2 (Scarb1, Alox15, and Mrc1), STAT6, and PPARγ activation [61]. TLR-mediated NF-κB activation can produce inflammatory mediators associated with M1 macrophages, but can also activate genetic programs essential for the resolution of inflammation and M2 polarization [62]. In addition, c-Jun N-terminal kinase (JNK) activation is required for M1 polarization of macrophages during obesity-induced inflammation and insulin resistance [63]. Other factors such as HIF-1α and HIF-2α are expressed differentially in M1 and M2 macrophages and regulate inducible NOS2 and arginase 1 expression, respectively [64].

More recently, microRNAs (miRNAs) have emerged as critical regulators of macrophage polarization. In particular, TLR signaling triggers the expression of a different set of miRNAs that regulate key molecules involved in macrophage polarization and affect the balance of pro- and anti-inflammatory responses [65]. miR-127-3p, miR-155-5p, miR-181a, miR-204-5p, and miR-451 were significantly upregulated in the presence of LPS and IFN-γ in murine BMDMs, while miR-125-5p, miR-143-3p, miR-145-5p, and miR-146a-3p were downregulated in M2-polarized BMDMs [47]. Moreover, miR-27a, miR-29b, miR-125a, miR-146a, and miR-155 were remarkably upregulated in polarized M1 macrophages, whereas miR-26a and miR-193b were upregulated in polarized M2 macrophages [66]. Therefore, the identification of specific miRNAs related to changes in macrophage polarization will increase our understanding of the molecular basis of disease progression and lead to the development of novel miRNA-targeted therapies.

### 3.2. Function of Liver Macrophages in NAFLD/NASH

Upon recognition of a foreign substance, KCs are activated and release a variety of inflammatory mediators, including cytokines (e.g., TNF-α, IL-1β, and IL-6), chemokines (monocyte chemotactic protein 1 (MCP-1)), macrophage inflammatory protein (MIP)-1α, MIP-1β, and RANTES [67]. All of these mediators may cause hepatic toxicity and liver function impairment. These factors regulate the phenotype of KCs as well as that of neighboring cells, such as hepatocytes, hepatic stellate cells (HSCs), liver sinusoidal endothelial cells, and other immune cells that traffic through the liver [41]. In parallel, cytokines and chemokines, which are secreted by KCs, lead to the recruitment of neutrophils, natural killer T lymphocytes, natural killer cells, and blood monocyte-derived macrophages to the liver [68,69].

KCs are involved in the control of inflammatory responses in NAFLD. During the early stages of the disease, hepatic macrophages expand rapidly and secrete cytokines and chemokines, such as IL-1β, TNF-α, MCP-1, and CCL5, contributing to paracrine activation of protective or apoptotic signaling pathways in hepatocytes, and the recruitment of other immune cells [70]. Moreover, these inflammatory mediators can lead to tissue damage in ischemia reperfusion, endotoxemia, acetaminophen-induced liver hepatotoxicity, and alcohol-induced liver steatosis [71,72]. KCs may also express immunoregulatory molecules, such as IL-10, and transforming growth factor beta (TGF-β). An unbalanced production of pro- and anti-inflammatory mediators by KCs can lead to liver injury [38,73].

Although hepatic insulin resistance is involved in the progression of NAFLD, multiple cytokines have been implicated in the pathogenesis of hepatic insulin resistance [43]. Obesity-induced KCs activation leads to the production of pro-inflammatory cytokines, which inhibit hepatocyte insulin signaling through a paracrine mechanism [74,75]. By regulating the oxidation of fatty acids, KCs increase lipid storage in hepatocytes during obesity, which results in hepatic insulin resistance [76]. This effect is triggered by inflammatory cytokines, such as TNFα, IL-6, and IL-1β [76]. Moreover, under certain conditions, alternative activation of KCs by IL-4/IL-13 has been found to ameliorate obesity-induced insulin resistance by regulating PPARδ [19,69], suggesting a beneficial role for M2-activated KCs in metabolic syndrome and NAFLD.

Fibrosis is a key feature of chronic liver inflammation and NASH, and activated macrophages exert a dual function in the orchestration of matrix deposition and remodeling [77,78]. HSCs, in response to damage, differentiate into myofibroblast-like cells, which produce extracellular matrix (ECM) components in pathology. HSCs and their progeny engage in bidirectional interaction with resident and recruited macrophages [79]. Depending on the context and specific activation signals, macrophages can exert dual functions on HSCs and ECM deposition [38]. Phagocytosis of dying necrotic cells and debris triggers the production of TGF-β by KCs and recruited macrophages [80,81]. On the other hand, phagocytosis of apoptotic hepatocytes and cholangiocytes has been shown to dampen the development of fibrosis [82]. IL-13 signaling, which polarize M2 macrophages, induces the production of TGF-β and its activation by matrix metalloproteinase-9 [83]. Moreover, monocytes/macrophages expressing chemokine receptors, such as C-C chemokine receptor (CCR)2, CCR1, and CCR5, are thought to interact with HSCs through TGF-β to promote fibrosis [84,85].

## 4. Carotenoids and NAFLD/NASH

Due to increasing prevalence and incidence, and a lack of established therapeutic interventions, NAFLD has emerged as one of the most important health problems worldwide. Therefore, increasing numbers of studies have focused on natural dietary compounds for the prevention and treatment of NAFLD. Antioxidant micronutrients, such as vitamins and carotenoids, exist in abundance in fruits and vegetables and defend against reactive oxygen species [86]. Carotenoids accumulate mainly in the liver and incorporate into lipoproteins for release into the circulation [87]. Ingested carotenoids may participate in an antioxidant defense system when free radical species in the liver are present at high concentrations, and these physiological functions of carotenoids could inhibit the development of liver dysfunction [88]. On the other hand, micronutrient antioxidants are severely depleted in the serum and liver tissue of patients with chronic liver diseases [89], and liver injury is associated with decreased antioxidant levels, particularly carotenoids [90]. Thus, micronutrient antioxidant deficiencies may contribute to the development of obesity and comorbidities, such as insulin resistance and NASH [91,92]. Recently, low serum concentrations of carotenoids, such as α-carotene, β-carotene and vitamin E, were shown to be associated with obesity [90,91,93]. Moreover, greater serum carotenoid levels are associated with lower serum ALT levels and a lower risk of developing NAFLD [94,95]. There is growing evidence for the use of vitamin E in the treatment of NASH [96]. Dietary α-tocopherol supplementation was recently found to attenuate LPS levels, and a MCD diet induced oxidative stress and inflammation-related responses in NASH in mice [97,98]. Importantly, carotenoids were found to be as potent as vitamin E in inhibiting lipid peroxidation [99]. However, carotenoid supplementation (β-cryptoxanthin and astaxanthin) has not been widely used as an antioxidant therapy in NASH treatments. The mechanism of action of carotenoids, including β-cryptoxanthin and astaxanthin, in NAFLD is unclear, but there is evidence they may work through multiple mechanisms, including antioxidant and anti-inflammatory effects [88,100], and regulation of M1/M2 macrophage polarization [101,102].

### 4.1. β-Cryptoxanthin

β-Cryptoxanthin is a xanthophyll carotenoid specifically found in the Satsuma mandarin (*Citrus unshiu* Marc.). β-Cryptoxanthin is readily absorbed and relatively abundant in human plasma, together with α-carotene, β-carotene, lycopene, lutein, and zeaxanthin [103,104,105]. Similar to other carotenoids, β-cryptoxanthin has antioxidant activity [106,107] and higher bioavailability than those of β-carotene in rodents [87]. Serum β-cryptoxanthin concentrations were found to be inversely associated with indices of oxidative DNA damage and lipid peroxidation [108]. Recent epidemiological studies showed that serum β-cryptoxanthin levels were inversely associated with insulin resistance risk and alcohol-induced increases in serum γ-glutamyltransferase levels in nondiabetic subjects and alcohol drinkers, respectively [104,105]. In addition, β-cryptoxanthin suppressed LPS-induced osteoclast formation in co-cultures of bone marrow cells and osteoblasts, and restored alveolar bone loss induced by LPS in mice [109]. Moreover, β-cryptoxanthin can accumulate in RAW264.7 cells and induce changes in the intracellular redox status, in turn regulating the immune function of macrophages [110].

**Figure 3:**
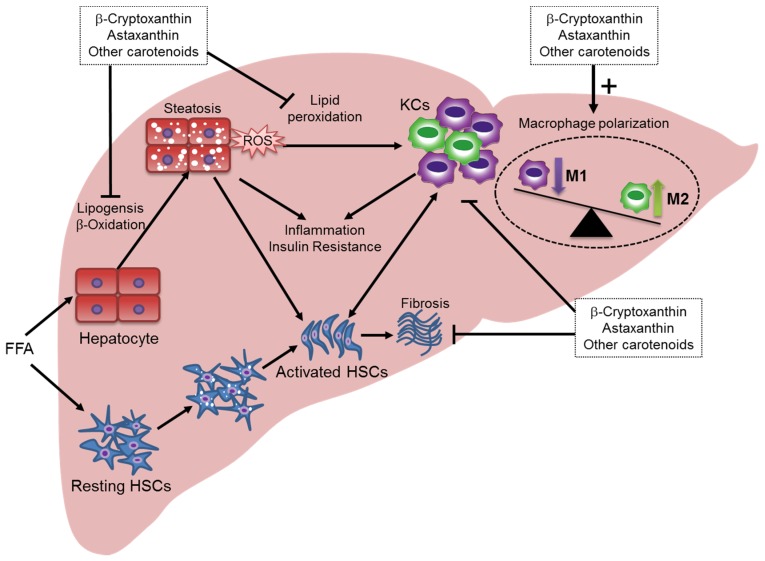
Schematic representation of the hepatoprotective effect of carotenoids on the progression of NAFLD/NASH. Carotenoids may improve NAFLD/NASH by inhibiting lipogenesis, β-oxidation of free fatty acids, inflammation, and HSC activation. In addition, apart from their common anti-oxidative and anti-inflammatory properties, carotenoids, such as β-cryptoxanthin and astaxanthin, can contribute to liver homeostasis by regulating the polarization of M1/M2 macrophages/KCs.

In our previous study, we found that β-cryptoxanthin prevented the development of NASH by attenuating fat accumulation, increases in KC numbers, activation of stellate cells, and fibrosis in mouse models of lipotoxicity-induced NASH [101,111]. Comprehensive gene expression studies have shown that β-cryptoxanthin is more effective in inhibiting the inflammatory gene expression changes that accompany NASH [111]. β-Cryptoxanthin downregulated the expression of genes associated with cell death, inflammatory responses, free radical scavenging, and infiltration and activation of macrophages, leukocytes, and T cells [111]. However, it showed little effect on the expression of genes related to the metabolism of cholesterol and other lipids [111]. Moreover, β-cryptoxanthin reversed pre-existing NASH in mice [101]. β-Cryptoxanthin inhibited lipid accumulation and peroxidation in the liver due to its strong anti-oxidative properties. Furthermore, β-cryptoxanthin reduced the accumulation of T cells and macrophages, and regulated the M1/M2 status of macrophages/KCs in the liver without affecting the recruitment of monocytes from the bone marrow [101]. Additionally, β-cryptoxanthin directly decreased LPS-induced M1 activation and augmented IL-4-induced M2 macrophage activation in vitro, suggesting macrophages may be directly targeted by β-cryptoxanthin (Figure 3) [101]. Therefore, strategies that inhibit M1 polarization and/or drive alternative M2 macrophage/KC activation may protect against inflammation, thereby halting NASH progression.

### 4.2. Astaxanthin

Astaxanthin is another xanthophyll carotenoid found in various microorganisms and marine animals, including salmon, crabs, and crustaceans [112]. Astaxanthin is well known for its strong antioxidant capacity [113]. It is 100–500-fold more effective than vitamin E at preventing lipid peroxidation. It has hepato-protective effects and can protect against inflammation, ulcers, cancer, neurodegeneration, diabetes, immune-system attacks, and cardiovascular disease [112,114]. Astaxanthin has been reported to inhibit carbon tetrachloride-induced lipid peroxidation and to increase glutathione (GSH) levels and superoxide dismutase (SOD) activity in rat liver [115]. Astaxanthin prevented diet-induced obesity and hepatic triglyceride accumulation and steatosis in mice [116,117]. Moreover, astaxanthin prevented and reversed the activation of mouse primary HSCs and suppressed the upregulation of fibrogenic genes by blocking TGF-β/Smad3 signaling [118,119]. In addition, astaxanthin ameliorated insulin resistance by protecting cells from oxidative stress [120]. Therefore, the use of astaxanthin as a nutritional supplement has increased significantly in recent years.

We recently compared the preventative and therapeutic effects of astaxanthin and vitamin E in a lipotoxic NASH mouse model [102]. We found that astaxanthin had significant preventative and therapeutic effects (Figure 3). Astaxanthin attenuated insulin resistance, hepatic lipid accumulation and peroxidation, stellate cell activation and fibrosis, and it decreased the proportion of proinflammatory or M1-type macrophages/KCs in diet-induced NASH. In addition, astaxanthin ameliorated simple steatosis, the early stage of NAFLD, in both genetically (ob/ob) and diet-induced obese mice. Finally, we demonstrated that astaxanthin has the potential to improve NASH in humans [102].

The different mechanisms of action of astaxanthin and vitamin E in NASH mouse models are intriguing, because both of these lipophilic antioxidants suppress hepatic lipid peroxidation to an equivalent extent. Collectively, these results suggest that astaxanthin is more effective at preventing and treating NASH than is vitamin E [102]. First, astaxanthin was superior to vitamin E at improving steatosis by suppressing lipid accumulation. Second, astaxanthin reduced inflammation and insulin resistance more potently than did vitamin E. Of note, these anti-inflammatory and insulin-sensitizing effects were associated with attenuated MAPK (JNK/p38 MAPK) signaling and NF-κB activation, decreased macrophage/KC and T cell accumulation, and enhanced alternative M2 macrophage activation in the liver. Finally, astaxanthin prevented and reversed hepatic fibrosis to a greater extent than did vitamin E. Our in vitro study demonstrated that astaxanthin can act directly on hepatocytes by decreasing lipid accumulation, enhancing insulin signaling and suppressing inflammatory signaling. Additionally, astaxanthin administration decreased M1 macrophage marker activation and increased M2 macrophage marker activation in RAW264.7 macrophages, indicating macrophages are also a direct target of astaxanthin [102]. Therefore, astaxanthin confers its beneficial effects by regulating macrophage homeostasis and may be a potential candidate for the prevention or treatment of insulin resistance and NASH.

### 4.3. Other Carotenoids and NAFLD

Other carotenoids such as lycopene, and β-carotene have also been demonstrated to exert a protective effect in NAFLD. Lycopene, a non-provitamin A carotenoid, is found at high concentrations in red fruits and vegetables such as tomatoes, red grapefruit, watermelon, and apricots. Dietary supplementation with lycopene reduces the risk of cancers in many organs, and has chemopreventive effects against other diseases, including nonalcoholic steatohepatitis-promoted hepatocarcinogenesis [121]. Lycopene also reduces the development of hepatic steatosis induced by a HF diet [122]. Significantly reduced plasma lycopene levels were observed in NASH patients, suggesting a potential interaction between low lycopene status and the development of liver diseases [123].

β-Carotene is the most widely distributed carotenoid in yellow-orange and dark green fruits and vegetables. Studies have shown the potential preventive and therapeutic effects of β-carotene on hepatic inflammation, fibrosis, and cirrhosis [88]. Dietary β-carotene supplementation was found to have a protective effect on liver damage [88].

## 5. Conclusions

NAFLD has become one of the most important chronic liver diseases in the world. Its association with obesity, type 2 diabetes mellitus, insulin resistance, metabolic syndrome, and progression to cirrhosis and HCC increases its clinical importance. NAFLD pathogenesis is very complex and may involve many mechanisms. A detailed understanding of the pathogenetic mechanisms is needed to develop new preventative and therapeutic strategies. Moreover, the treatment of NAFLD patients should be based on a global approach, and it should address not only insulin resistance and metabolic syndrome, but also strategies to reduce oxidative stress, dyslipidemia, and cardiovascular risk. Although insulin resistance and increased oxidative stress are believed to be major risk factors for the progression to NASH many agents, including insulin sensitizers, have been evaluated with disappointing results in the management of NASH. Carotenoids, which are natural antioxidant compounds possessing anti-inflammatory properties, appear to be beneficial in the prevention and treatment of NAFLD. Our recent studies provide evidence that dietary β-cryptoxanthin or astaxanthin can prevent and reverse the progression of NASH in mice. Other carotenoids, including lycopene and β-carotene, may also help improve NAFLD progression.

Macrophages play an important role in the pathogenesis of oxidative stress, insulin resistance, and NAFLD. Therefore, new therapies for the treatment of inflammation and insulin resistance may involve strategies that modify macrophage polarization by either restraining M1 activation or driving M2 activation. In addition to their common effects, carotenoids, such as β-cryptoxanthin and astaxanthin, may contribute to liver homeostasis by directly regulating macrophage polarization, thereby halting the progression of NASH. Future investigations are warranted to elucidate the precise mechanisms mediating the preventative and therapeutic effects of carotenoids in NAFLD.

## Abbreviations

The following abbreviations are used in this manuscript:

NAFLD	Non-alcoholic fatty liver disease
NASH	Non-alcoholic steatohepatitis
HCC	Hepatocellular carcinoma
KC	Kupffer cell
HSC	Hepatic stellate cell
TLR	Toll-like receptor
LPS	Lipopolysaccharide
IFN-γ	Interferon-gamma
IRF	Interferon Regulatory Factor
STAT	Signal Transducer and Activator of Transcription
SOCS	Suppressor of Cytokine Signaling
PPAR	Peroxisome Proliferator Activated Receptor
KLF4	Krüppel-Like Factor 4
ECM	Extracellular Matrix
CCL	C-C chemokine ligand
CCR	C-C chemokine receptor
